# The economics of the UN Sustainable Development Goals: does sustainability make financial sense?

**DOI:** 10.1007/s43621-022-00088-5

**Published:** 2022-06-20

**Authors:** Walter Leal Filho, Maria Alzira Pimenta Dinis, Salvador Ruiz-de-Maya, Federica Doni, João Henrique Eustachio, Julia Swart, Arminda Paço

**Affiliations:** 1 Department of Natural Sciences, Manchester Metropolitan University, All Saints Building, Oxford Road, Manchester, M15 6BH UK; 2 UFP Energy, Environment and Health Research Unit (FP-ENAS), University Fernando Pessoa (UFP), Praça 9 de Abril 349, 4249-004 Porto, Portugal; 3 Marketing Department, University of Murcia, Campus de Espinardo, 30100 Murcia, Spain; 4 Department of Business and Law, University of Milano-Bicocca, Via Bicocca degli Arcimboldi, 8, 20216 Milan, Italy; 5 School of Economics, Business Administration and Accounting at Ribeirão Preto, University of São Paulo (USP), Avenida dos Bandeirantes 3900, Ribeirão Preto, SP 14040-905 Brazil; 6 Utrecht School of Economics, Utrecht University, Kriekenpitplein 21-22, 3584 EC Utrecht, The Netherlands; 7 NECE-UBI (Research Centre for Business Sciences), Universidade da Beira Interior, R. Marquês D’Ávila e Bolama, 6201-001 Covilhã, Portugal; 8 Research and Transfer Centre “Sustainable Development and Climate Change Management”, Hamburg University of Applied Sciences, Ulmenliet 20, D-21033 Hamburg, Germany

**Keywords:** Sustainable Development Goals (SDG), Economic impact, Investment, SDG financing

## Abstract

The implementation of the UN Sustainable Development Goals is a global priority, but one whose full implementation is vulnerable to the high costs associated with it. This raises the question: does the implementation of the SDGs make financial sense? This article addresses this question and outlines the need to raise awareness of the economic benefits of implementing the global goals. Further, it presents and discusses the main financial gaps to achieve the Sustainable Development Goals by 2030.

## Introduction

The 17 United Nations Sustainable Development Goals (SDGs) were designed to create a vision for achieving a sustainable future. An international framework was formed to help countries with their development efforts. Furthermore, it is intended to create a sense of accountability and pressure that allows positive progression toward sustainable development [1]. The SDGs target developing or less developed countries by urging richer nations to support programmes in the less deprived or developed regions [2, 3].

More specifically, the SDGs were designed to, among other things, provide a basis to end poverty, eradicate hunger, protect the planet, and improve the quality of life in the world, ensuring a balance is achieved between social, economic, and environmental sustainability [4]. These challenges are more pressing in the least developed countries, which are precisely those countries lacking the (financial) resources to implement the necessary changes. In this article, we analyse the importance of financial instruments for achieving the SDGs, and we assess the gaps in attaining the SDGs in a timely manner. The costs to achieve the SDGs, the challenges faced by investors, and the various factors related to sustainable investment are taken into consideration. Having this clear understanding of the economic and financial aspects related to pursuing the SDGs is fundamental for tracing the plans for achieving them within the 2030 frame.

## The UN Sustainable Development Goals and their economic aspects

The implementation of the SDGs has deep economic implications. The urge for achieving the SDGs by 2030 implies that more attention should be placed on the analysis and assessment of the costs of implementing the SDGs and the (intertemporal) costs of not pursuing them [5–8]. Thus, several organisations have been carrying out costing exercises regarding the implementation of the SDGs, as well as estimates of the global resources needed to achieve them. The results, however, hardly allow comparisons [9].

For instance, the 2014 World Investment Report estimated that USD$5 trillion to USD$7 trillion per year, between 2015 and 2030, would be necessary to globally achieve the SDGs, where USD$3.3 trillion to USD$4.5 trillion per year would be dedicated to the developing countries, mainly for basic infrastructure, food security, climate change policies, health, and education [10]. One of the most prominent studies carried out later by OECD [11] defends that the policies and resources of OECD countries (e.g., taxation, investment, fees, transference of funds) can have a huge impact on enabling more sustainable financing. However, if it is then take into account that around 80% of global financial assets (USD$ 379 trillion) are kept in OECD countries, the governments of low-income developing countries will need significant growth in fiscal income and international cooperation to enable them to finance the costs needed to implement the SDGs [12].

In addition to this complexity, with the unexpected event of the pandemic caused by COVID-19, the investments needed to implement the SDGs have been compromised. This is problematic, because even before the pandemic emerged, the progress to attain the targets was already meager, facing several constraints [13, 14]. Before the crisis triggered by the pandemic, most countries were already struggling to finance the 2030 Agenda, with a financial gap of USD$ 500 billion for low-income countries and USD$ 2 trillion for other developing countries [15]. Furthermore, McKinsey Global Institute [16] estimates that countries should spend about US$3.3 trillion annually to close the infrastructure gap by 2030 (deficit of US$0.8 trillion), which comprises the costs for basic and local infrastructure in high, middle, and low-income countries.

But the economic dimension of implementing selected SDGs, as highlighted by Black Rock [17], needs to include the estimated cost of inaction (as a share of global GDP). Biodiversity loss, for example, generated an estimated cost of USD$ 10–31 trillion per year or 11% to 36% of global GDP in the period 1997 to 2011 [18]. Moreover, this cost of inaction is not limited to the SDGs that are not progressing at the required speed to meet the deadline (2030), which means that it is necessary to treat SDGs as a network and not as stand-alone targets. The elimination of this cost of inaction requires, in addition to funds, the engagement of different stakeholders—the private sector, official development assistance, international financing institutions, civil society, and philanthropies.

The Addis Ababa Agenda Action argues the need for more money to finance public services for achieving the SDGs [19]. The least developed countries, in particular, need a strict commitment of the public sector to stimulate the mobilisation of financial resources. International financial institutions can reduce the perceived risks, while national policies can incentivise the alignment of financial systems to long-term sustainable development and increase access to finance. To better align private sector incentives and practices with SDGs that foster long-term quality investment, some proposals have introduced a consistent framework [15], a SDG Industry matrix [19], and the identification of infrastructure assets that are likely to have certain outcomes in line with the SDGs [20], or the understanding of SDGs through the financial materiality lens [17].

Few studies have also analysed specific SDGs. Prakash et al. [21], for example, evaluated the costs to achieve SDG 11, which relates to sustainable cities and communities and identified difficulties in its assessment due to the complexity and scope of the urban systems. Despite these difficulties, further research should be carried out to understand the costs of reaching SDG 11, given the need for smart, sustainable, and efficient cities, and the forecast that about 60% of the population will be living in urban areas by 2030 [21]. In turn, Hutton and Varughese [22] analysed SDG 6 (clean water and sanitation) and estimated that an investment of US$13.8 to $46.7 billion per year is necessary to achieve two out of the eight targets (6.1—Safe and affordable drinking water and 6.2—Sanitation and hygiene for all), predicting great difficulties in most low and middle-income countries, as well as in high-income countries with low WASH (Water, Sanitation, and Hygiene) coverage. Concretely, resources need to be moved to basic sanitation and hygiene in countries and areas where this gap is larger (for instance, urban areas account for 70% of the costs to achieve basic WASH). Regarding SDG 3 (good health and well-being), Stenberg et al. [23] analysed 67 countries with different health systems and estimated that an additional $274 billion in spending on health is needed per year by 2030 to make improvements in this goal. In a more ambitious scenario, the total healthcare expenditure would raise around $271 per person. Notwithstanding the increases in health investment, a financing gap of $20–54 billion per year is forecasted by the authors. Development Initiatives (DI) [24] analysed the gaps in existing estimates for SDG 3 and SDG 4 (quality education) and found that the main problems were ‘the costs of scaling up to cover the full population; the higher unit costs of reaching the most marginalised people and places; the costs of some health interventions; the costs of reaching people affected by future crises; the full costs of moving to more equitable financing models.

## Overcoming financial gaps for SDG attainment at the country scale

**Fig. 1:**
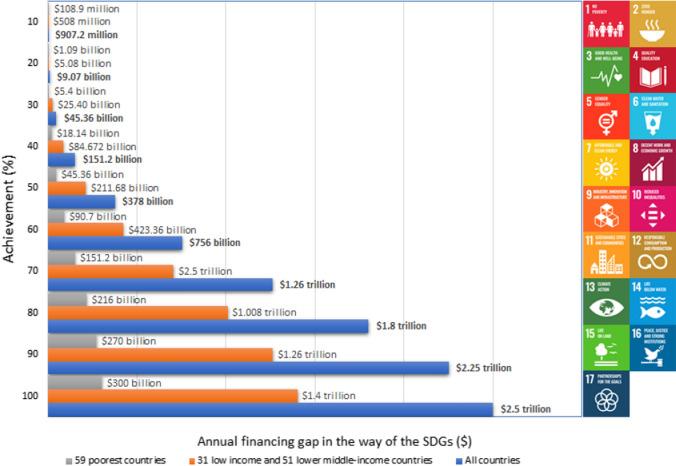
Financial gaps to achieve SDGs. Source: based on data from United Nations (2019) and UNA-UK (2019)

Figure 1 illustrates the financial gaps hindering the SDGs to be achieved as a whole, according to data from the United Nations [25] and UNA-UK [26]. The graphics allow us to compare the needs in the three reported cases, i.e., the 59 poorest countries; the 31 low-income and 51 lower-middle-income countries; and all countries which, together need to further advance the SDGs. The values presented report the achievement percentage in each case. The annual SDG funding gap lies between $1.4 to $3 trillion and between $300 to $528 billion for the ‘low-income and lower middle-income and the ‘poorest’ countries, respectively. However, for ensuring clarity, only the lower limits have been projected in Fig. 1 for both cases. The values mentioned above reflect the existing financial gap in case 100% achievement of SDGs is to be fulfilled by 2030.

The implementation of the SDGs is a global priority, and the role of economic instruments and sustainable financing options became essential in overcoming the costs associated with implementing the 2030 agenda through bridging the funding gaps for socioeconomic and environmental challenges [27].

In particular, great attention has been paid to sustainable financing and investments [28]. Some examples of these instruments are private investments, foreign direct investments, funding sources for sustainable development, sustainable bonds, and other sources of capital from local, federal, and international sources [27. 28]. In addition, economic and policy instruments such as taxes, tax exemptions, or auctions could mitigate the negative externalities towards a more sustainable society. Table 1 shows a summary of the main recent country-specific initiatives undertaken for green and sustainable financing.

**Table 1:** Country-specific initiatives for green sustainable financing

Country/sector	Reference	Description
Western Balkan countries	Lukšić, et al. [29]	Use of both historical and innovative instruments for financing sustainable green development
G7 countries	Yang et al. [30]	Green finance is observed as a key determinant of sustainable performance, measured through Environment Social & Governance (ESG) indicators)
Africa	Aust et al. [31]	Foreign Direct Investments (FDI) can benefit SDGs scores in developing economies
United Nations	Financing for Sustainable Development Report 2022. Inter-agency Task Force on Financing for Development [8]	The report recommends urgent measures to financing gaps and rising debt risks, to align financing flows with sustainable development, and to improve information transparency
European Council	EU Long term budget 2021–2027 [32]	The EU commitment to the SDGs includes an unprecedented target of green spending by allocating 30% of the total budget to fight climate change

### CSR in the private sector and SDGs

The relationship between the private sector’s CSR initiatives and SDGs is largely recognized and can generate positive financial effects [33–36]. For example, Schramade [37] explored the investment opportunities of SDGs by identifying two kinds of reasons for investing in SDGs: returns to society and returns to shareholders which evidences some differences among SDGs in terms of estimated amounts of opportunities. More recently, a study carried out by Consolandi et al. [38] offered a methodology for measuring the contribution of health care companies to achieve SDG 3 linking the goal’s targets with the SASB’s generic ESG issues by adopting the financial materiality perspective. Nevertheless, a scarce engagement with the SDGs for organizations may create a process of “SDGs washing” [39], this is, a process in which firms use SDGs to market their positive contribution to achieving some objectives while neglecting their negative impact on others, or it can negatively affect the “sell” recommendations of financial analysts [40].

## Implementing the SDGs: investment and cost of not implementing

Achieving the SDGs should not, however, be seen only from a cost perspective, but also as an investment. The SDGs can open up relevant growth opportunities in terms of market opportunities, estimated at USD$ 12 trillion in four relevant economic systems, i.e., food and agriculture, cities, energy and materials, and health and well-being. This global economic advantage can be higher by considering the whole economic system and the increase in labour and resource productivity [17].

**Fig. 2:**
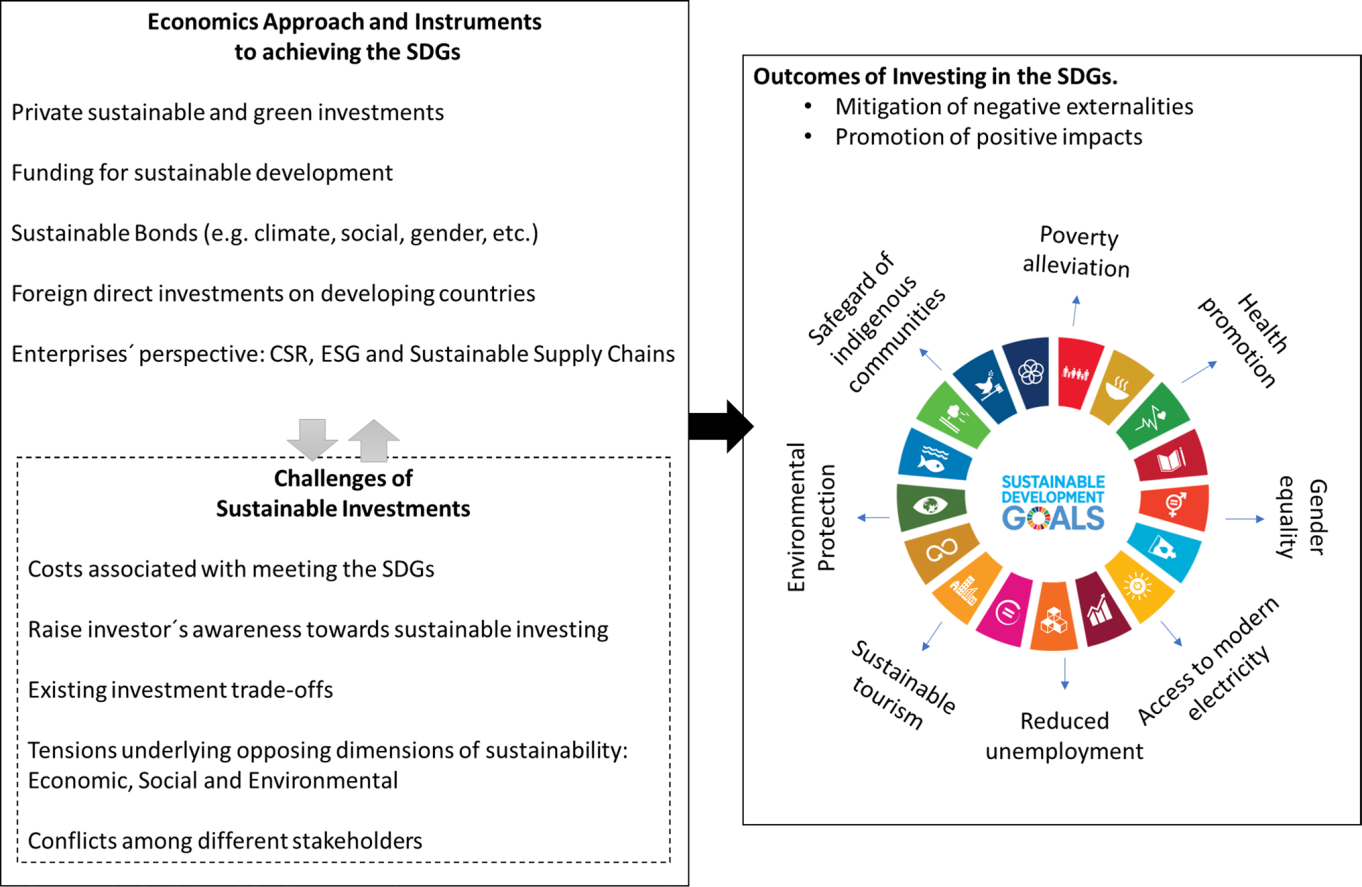
Outcomes of investing in the SDGs

However, despite the several existing approaches to deal with sustainability challenges, there is a latent need to perceive the positive economic aspects of sustainability when implementing the SDGs and to overcome the costs involved. The economic perspective with several instruments—such as sustainable investments, accountability and transparency mechanisms [11]—play an essential role in helping societies meet the SDGs, either by reducing the negative externalities or by fostering the positive ones. Figure 2 synthesises this perspective by illustrating how economic approaches and sustainable investments and funding make sense for sustainability promotion. The figure illustrates, for example, that the SDGs promote more justice around the world, which has an economic benefit (e.g., less money devoted to security and prosecution processes). Many other arrows can be added to Fig. 2, which all lead to a positive economic impact.

## Conclusion

As this article has shown, although sustainable investments make sense for pursuing the 2030 Agenda, there are still challenges to overcome, such as the costs associated and the lack of investors' awareness in making sustainable investments, changing the old belief that sustainable investing is not profitable. In addition, recent research on the paradox of corporate sustainability points to the complexity of dealing with sustainable investing, as it is a decision-making process that happens in several contexts that involve several trade-offs, competing interests among various stakeholders, and even conflicting objectives when considering the dimensions of sustainability. Despite these challenges, there is an urge for researchers and organisations to deepen their understanding of the mechanisms to maximise sustainable investing and of how it can become common and not an exception among investors and other stakeholders so that the SDG financing gap can be addressed, benefiting the people and the planet [11]. In summary, the social cost of not achieving the SDGs should urge us to join efforts and make use of synergies to reach a better world for current and future generations.

## Data Availability

Not applicable.
